# Design and Implementation of an Ultra-Low Resource Electrodermal Activity Sensor for Wearable Applications ‡

**DOI:** 10.3390/s19112450

**Published:** 2019-05-29

**Authors:** Gunnar C. Pope, Ryan J. Halter

**Affiliations:** Thayer School of Engineering at Dartmouth, Dartmouth College, Hanover, NH 03755, USA; gunnar.c.pope.th@dartmouth.edu

**Keywords:** electrodermal, wearable, biosensor, data compression, signal processing, low-resource

## Abstract

While modern low-power microcontrollers are a cornerstone of wearable physiological sensors, their limited on-chip storage typically makes peripheral storage devices a requirement for long-term physiological sensing—significantly increasing both size and power consumption. Here, a wearable biosensor system capable of long-term recording of physiological signals using a single, 64 kB microcontroller to minimize sensor size and improve energy performance is described. Electrodermal (EDA) signals were sampled and compressed using a multiresolution wavelet transformation to achieve long-term storage within the limited memory of a 16-bit microcontroller. The distortion of the compressed signal and errors in extracting common EDA features is evaluated across 253 independent EDA signals acquired from human volunteers. At a compression ratio (CR) of 23.3×, the root mean square error (RMSErr) is below 0.016 μS and the percent root-mean-square difference (PRD) is below 1%. Tonic EDA features are preserved at a CR = 23.3× while phasic EDA features are more prone to reconstruction errors at CRs > 8.8×. This compression method is shown to be competitive with other compressive sensing-based approaches for EDA measurement while enabling on-board access to raw EDA data and efficient signal reconstructions. The system and compression method provided improves the functionality of low-resource microcontrollers by limiting the need for external memory devices and wireless connectivity to advance the miniaturization of wearable biosensors for mobile applications.

## 1. Introduction

Advances in miniaturizing and increasing computational functionality of wearable biosensors have revolutionized the delivery of modern healthcare services by providing pragmatic and timely solutions that are scalable outside of the hospital. Embedded microcontrollers (MCUs) play critical roles in wearable biosensor systems, providing high-performance digital signal processing (DSP) capabilities at ultra-low power consumption due to efficient usage of their single core central processing units (CPUs). The gains in power efficiency and compact size come at the cost of low internal storage capabilities (∼1 kB to 512 kB range) and lower processing capabilities when compared to larger, multi-core microprocessors and system-on-a-chip (SoC) solutions.

Designers of wearable, ultra-low-power or energy harvesting biosensors must address the challenge of maximizing sensor performance while minimizing physical size, weight, and power consumption to create unobtrusive wearable biosensors [1,2]. High-quality recording of physiological signals is commonly associated with high data rates and large storage requirements for embedded systems, often requiring the use of external memory devices, such as microSD cards, peripheral memory chips (EEPROM, Flash, etc.), or wireless transmission to a phone or other off-system device.

Advanced signal processing techniques can be applied on microcontrollers in real time to leverage their advantages and create the next generation of autonomous and energy harvesting biosensors. Significant improvements in miniaturization, data-rate, power, and latency can be achieved by extending the capabilities of modern microcontrollers to include on-node processing, data compression, machine learning, and energy harvesting by reducing system operating requirements [3,4,5,6,7,8]. Deepu et al. [8] suggest that system level power can be improved with the use of on-chip lossy compression and storage but notes that storing data in memory for future use has the additional challenges of referencing real time.

The approach described here enables low-resource microcontroller to be used as a complete system for continuously recording physiological data, specifically the electrodermal activity (EDA) signal, by applying advanced wavelet signal decomposition techniques, data compression, and data storage entirely within a 16-bit microcontroller having only 64 kB of total memory. These techniques can enable continuous on-chip physiological recordings >2 h without requiring wireless data transmission or external memory chips. The signal processing methods described below were designed to produce high-quality recordings of EDA, but could be extended to other physiological signals of similar bandwidth. Preliminary developments of this approach were described in a earlier conference proceedings [9]. Here, we expand upon this earlier work and present a more efficient compression algorithm along with detailed implementation strategies. The novel contributions of this article are:

1. An optimized embedded EDA compression algorithm for 16-bit MCU architectures that improves upon the initial work from Pope and Halter [9].
2. A compression performance comparison of this low-resource EDA compression method to a recent compressive sensing (CS) method from Chaspari et al. [10].
3. Quantification of the compression distortion on common tonic and phasic EDA signal features frequently used in affective computing research.
4. Demonstration of improved power performance of compressing and storing EDA signal within a single 16-bit microcontroller as compared to methods requiring external memory.

To the best of our knowledge, this is the first publication to tailor an on-board wavelet compression algorithm to the EDA signal and compare its performance to other CS-based methods. Additionally, this is the first detailed description of an EDA compression algorithm designed for a embedded sensor implementation. After an overview of EDA, wavelet transformations, and data compression methods, the rest of the manuscript is structured to present the methods, results, and applications of the designed system.

### 1.1. Electrodermal Activity

**Figure 1:**
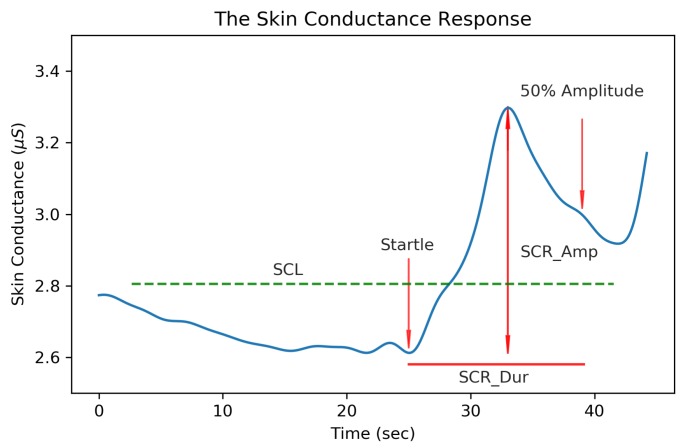
Features of the skin conductance response (SCR). The skin conductance level (SCL) was computed as the average skin conductance (SC) value across a 64 s window, SCR_Dur is the time from the beginning of the SCR to the 50% amplitude level, and SCR_Amp is the amplitude from the minimum to the maximum of an SCR.

EDA is a physiological phenomena that refers to electrical variations occurring on the surface of the skin due to changes in sweat secretion. The electrodermal response (Figure 1) is exclusively activated by the sympathetic nervous system [11], making it a leading biosignal to monitor psychological or physiological arousal of the autonomic nervous system. Because of this relationship, EDA monitoring has been used for multiple applications including evaluating anxiety and stress [12,13,14,15], detecting the orienting response [16], providing biofeedback for epilepsy mitigation [17], recognizing emotional states [18,19], and many more. Some applications rely on acute, short-term EDA changes to impact the user (e.g., identification of epileptic events), while, in others, long-term trending is more important (e.g., long-term anxiety and stress monitoring.)

Measures associated with EDA are typically grouped into two classes of tonic and phasic features. Tonic EDA measures, relating to the slowly varying skin conductance level, have been “...long established as the most frequently used indicator of arousal in psychophysiological research” [16]. The tonic features shown in Figure 1 are the average skin conductance level (SCL), the standard deviation of the EDA signal (EDA_Std), the signal maximum amplitude (SC_Max) and minimum (SC_Min). Phasic features relate to the rapid fluctuations of skin conductance (SC) in response to a stimulus and have been helpful in evaluating stress [12,13], anxiety [14,15], the orienting response [16], and applications related to emotional sensing [18,19]. These skin conductance responses (SCRs) have both an SC amplitude (SCR_Amp), measured from the trough to peak of a SC pulse, and a duration (SCR_Dur), measured as the time between the onset of an SCR to the point when the SC level reaches 50% of its peak amplitude. The area under the curve of an SCR (SCR_AUC) is measured according to SCR_AUC = 1/2 SCR_Amp × SCR_Dur to provide a relative estimate of an SCR size. Studies involving EDA are commonly interested in the psychophysiological response to emotional stimuli which can vary depending on the measurement location. Sweat gland concentrations are not evenly distributed across the body, with the largest densities being measured on the soles of the feet (620/cm${}^{2}$), the forehead (360/cm${}^{2}$), the palms and cheeks (300/cm${}^{2}$), and the thighs (120/cm${}^{2}$) and not all measurement locations are equally responsive to emotional stimuli [16,20]. In 2012, Van Dorren et al. [21] compared 16 different measurement locations for sensing emotional sweat on the body and report the mean amplitude and standard deviation of these locations. This study confirmed that emotional sweating can be recorded at measurement sites other than the palmar and plantar surfaces but often at a reduced amplitude and activation levels. In this study, EDA is measured at the wrist since the form factor of wrist-worn biosensors are commonly accepted in commercial devices and bracelets provide good mounting surfaces for attaching electrodes to the skin. Ideally, this proposed system could be further miniaturized to record EDA at the palmar surfaces (where SCR activity is more prominent) without being obtrusive or encumbering daily activity.

### 1.2. Wavelet Transformations

**Figure 2:**
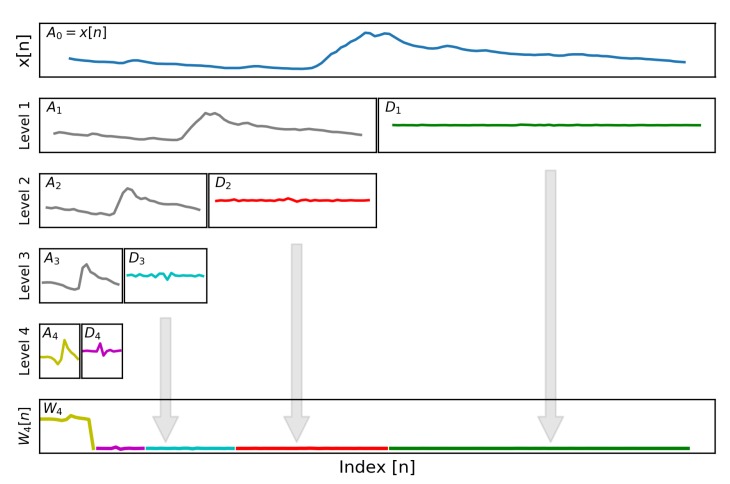
The physiological signal, x[n], has a sparse representation in the wavelet domain, $W_{4}\left[n\right]$, through the application of a multilevel discrete wavelet transformation. This technique was applied within the microcontroller to allow for effective signal compression. Each level of the transformation represents low-passed and band-passed versions of the original signal at different scales.

The discrete wavelet transform (DWT) has multiple applications in bioengineering that include artifact removal, signal de-noising, event detection, and signal compression [22] due to its ability to capture and localize temporal variations of a signal at a variety of scales [23]. The wavelet transformation has specifically been implemented on microcontrollers to detect respiration from a photoplethysmogram (PPG) signal [24], perform QRS-wave peak detection and denoising of an electrocardiogram (ECG) signal [25], improve energy performance of transmitting ECG and electromyogram (EMG) data wirelessly [26], and compress images and reduce transmission bandwidth in wireless sensor nodes [27]. The DWT, shown in Equations (1) and (2), is useful for its unique time-scale representation of physiological signals that are created during the convolution of an input signal, x[n], with a wavelet basis, h[n]. The Daubechies wavelet family is a popular wavelet family for physiological signal processing and is characterized by a set of scaling and wavelet coefficients that have low-passing and band-passing characteristics similar to that of quadrature mirror filters:

(1) $$\begin{array}{cc} A\left[n\right]=\left(x*h_{0}\right)\left[n\right] & =\sum_{k=-\infty }^{\infty }x\left[k\right]h_{0}\left[n-k\right], \end{array}$$

(2) $$\begin{array}{cc} D\left[n\right]=\left(x*h_{1}\right)\left[n\right] & =\sum_{k=-\infty }^{\infty }x\left[k\right]h_{1}\left[n-k\right], \end{array}$$

where A and D are the scaling and wavelet coefficient vectors and $h_{0}$ and $h_{1}$ are the Daubechies (db3) scaling and wavelet coefficients, respectively. This convolution decomposes the input signal into a series of time-scale or time-resolution representations useful in signal analysis. If the DWT is applied recursively, a multiresolution decomposition is possible. The coefficients of the multilevel discrete wavelet transform (ML-DWT) algorithm at a given transformation level, *L*, are calculated as:

(3) $$A_{L+1}\left[n\right]=\sum_{k=0}^{5}h_{0}\left[k\right]A_{L}\left[2n+k\right],$$

(4) $$D_{L+1}\left[n\right]=\sum_{k=0}^{5}h_{1}\left[k\right]A_{L}\left[2n+k\right],$$

where $A_{L}$ and $D_{L}$ are the approximation and detail coefficient vectors at a specific level and $h_{0}=h_{0}\left[0\right],h_{0}\left[1\right],...,h_{0}\left[5\right]$ and $h_{1}=h_{1}\left[0\right],h_{1}\left[1\right],...,h_{1}\left[5\right]$ are the Daubechies (db3) scaling and wavelet coefficients, respectively, with $\left|\right|h_{0,1}{\left|\right|}_{2}^{2}=1$. This algorithm recursively applies the DWT to produce a multiresolution decomposition of the EDA signal, as shown in Figure 2. Equations (3) and (4) produce a dyadic wavelet transformation, where $A_{L+1}$ and $D_{L+1}$ are each half the length of the previous approximation coefficient vector, $A_{L}$. For each coefficient vector $A_{L}$, the next level transformations, $A_{L+1}$ and $D_{L+1}$, represent low-passed and band-passed versions of $A_{L}$, respectively, providing the multiresolution decomposition of the EDA signal in the wavelet domain. Additional details on the wavelet transform and the filtering effects of the multiresolution decomposition can be found in [22,28,29,30]. The complete ML-DWT of the EDA signal can be represented as a 1D array, $W_{4}$, by concatenating the highest level approximation vector, $A_{4}$, along with all detail coefficients vectors, $D_{4}-D_{1}$, as shown Figure 2. These coefficients are all that is needed to fully reconstruct the original input signal, x[n].

#### Data Compression

Various methods for compressing physiological signals have been developed over the past 20 years to improve sensor size and power although few have been applied in the field of electrodermal activity. Data compression methods can often be separated into lossless or lossy compression. Lossless compression methods create perfect reconstructions from compressed data but are often processing intensive and challenging to implement on resource-constrained devices [31]. In contrast, lossy compression methods create reconstructions which are approximations to the original data and enable large levels of data compression at the expense of signal distortion. Since wearable biosensors are often resource-constrained devices, lossy compression methods are attractive for their ease of implementation and compression performance on embedded devices if the distortion can be tolerated [5,31]. Traditional data compression methods commonly involve sampling a physiological signal above the Nyquist rate, transforming the data into a sparse domain (having many near-zero values), and compressing the data using a certain threshold criteria. Data compression has been used in ECG and EMG applications to improve energy performance of transmitting data wirelessly [26], and to compress images and reduce transmission bandwidth in wireless sensor nodes [27].

Compressive sensing (CS) is a new and promising field of research that is being applied to data compression on wearable devices. The fundamentals of CS presented by Candes, Tao, and Donoho [32,33] use a compressed sampling process that randomly sub-samples a signal below the Nyquist rate and, by leveraging sparsity and well-suited basis function, creates an approximation to the original signal by solving an underdetermined system of linear equations. Compressive sensing methods are beneficial when minimal on-board processing and data storage is desired. Chen et al. [34] provide one of the first implementations of an on-chip compressive sensing device for recording EEG signals stating that their method is agnostic to the input signal type assuming that the signal is sparse. This assumption is non-trivial in regards to the EDA signal since it is characterized by large shifts in baseline SCL and SCRs over time. A variety of studies have examined different models and basis functions to characterize the EDA signal in a sparse domain, such as sigmoid-exponential function [35], biexponential impulse function [36], dynamic casual model using variational Bayesian decomposition [37], and a multi-level (db3) wavelet transformation [9]. A recent paper by Chaspari et al. [10] is the only study to our knowledge presenting a CS-based method to compress the EDA signal. Their study uses EDA-specific dictionaries of tonic and phasic atoms to represent the long-term and short-term fluctuations of the EDA signal and the reconstruction is achieved by solving the CS minimization problem using orthogonal matching pursuit (OMP) techniques. Although this technique was not implemented on a wearable device, the results from this study provide a helpful performance comparison between our on-board approach and these CS compression methods.

## 2. Materials and Methods

### 2.1. System Description

**Figure 3:**
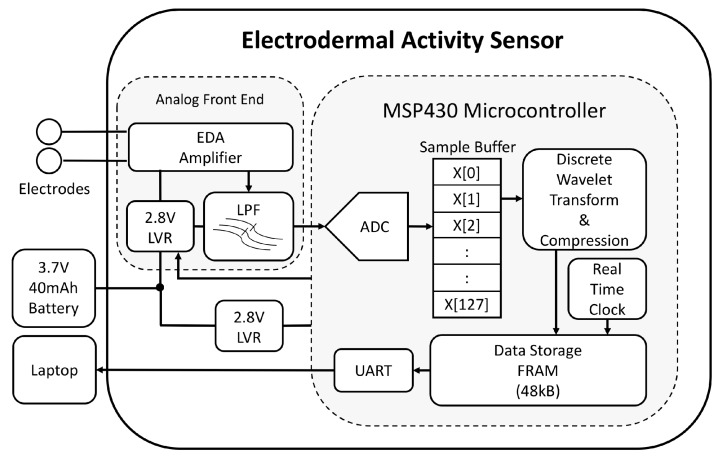
System diagram of developed electrodermal activity sensor. *ADC* = analog-to-digital converter, *EDA* = electrodermal activity, *LPF* = low pass filter, *LVR* = linear voltage regulator, *FRAM* = ferroelectric random access memory, *UART* = universal asynchronous receiver-transmitter serial communication protocol, *X* = skin conductance samples.

**Figure 4:**
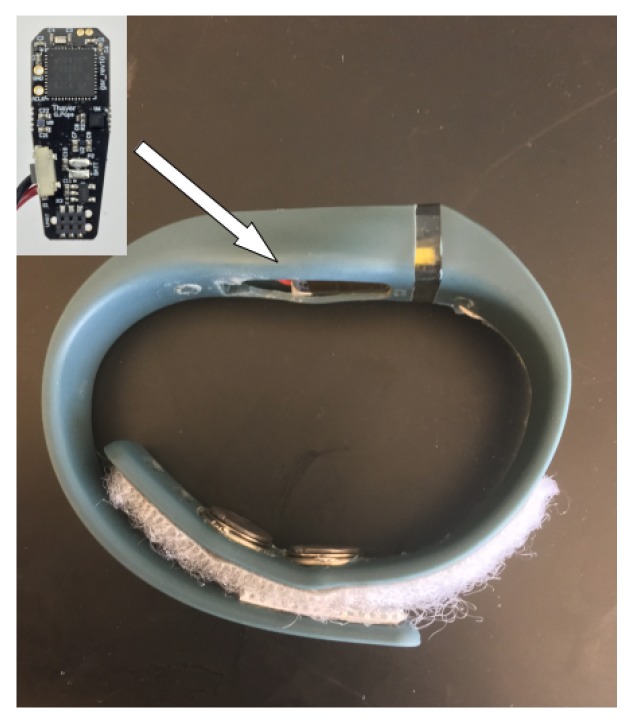
The complete EDA sensor assembly is shown above. Two, Ag/AgCl electrodes (1 cm diameter) were fastened along the inside of the wristband and measured electrodermal activity at the ventral wrist. Velcro strips were used to provide flexible sensor adjustment and to ensure a tight fit. The footprint of the printed circuit board (PCB) shown is 3.73 cm${}^{2}$. The system additionally has a three-axis accelerometer, skin temperature sensor, and event marker, although they were not used in this study.

A low resource biosensor was designed to continuously measure the electrodermal activity signal for multiple days using only a low-resource 16-bit microcontroller for signal processing and data storage. The sensor system (Figure 3) is composed of an analog front end (AFE) to amplify and filter the EDA signal and a digital signal processing back end within the MCU to compress and store the EDA signal. All sensor electronics were designed to fit within a small Fitbit Flex style wristband (Figure 4). Data are downloaded from the device to a laptop using the universal asynchronous receiver-transmitter (UART) protocol for serial communication.

### 2.2. Analog Front End

The AFE is composed of an EDA amplifier followed by a 4th order low pass filter (LPF). The LPF (Appendix B) was designed to pass the EDA signal from 0–1 Hz since the frequency response of sympathetic activities for physical and cognitive stress fall below 0.25 Hz [38,39]. The EDA amplifier measures electrodermal activity at the ventral wrist using a single op-amp (Analog Devices, AD8603, Norwood, MA, USA) topology, found in [16]. This topology (Appendix A) uses a quasi constant-current model for skin conductance measurement. The 1 cm diameter Ag/AgCl electrodes (Thought Technologies LLC, Montreal, QC, Canada) are replaceable and snap into place on the inside of the wristband shown in Figure 4. The electrodes are mounted using 1.5 cm center-to-center spacing. The accuracy and sensitivity of the device is shown in the Results section.

### 2.3. Microcontroller

This system uses an ultra-low power, 16-bit embedded microcontroller (Texas Instruments, MSP430FR5969, Dallas, TX, USA) for digital signal processing and on-board data storage of the EDA signal. The MSP430 is regulated at 2.8 V with a low-noise linear voltage regulator (Texas Instruments, LP5907, Dallas, TX, USA) supplied by a rechargeable 3.7 V, 40 mAh lithium ion battery (Sparkfun, PRT-13852, Niwot, CO, USA). We used an interrupt-based embedded code model [40] to set the MSP430 in ultra low-power mode between intervals of sampling and signal processing. The EDA signal is sampled at 2 Hz from the AFE using a 12-bit ADC on the MSP430. EDA data are stored into a sample buffer before being compressed. All signal compression is conducted in real time and compressed EDA data are saved into the lower 48 kB of non-volatile memory on the MSP430, as shown in Figure 3. Timing for the real-time clock (RTC) and analog-to-digital converter (ADC) is maintained using an external crystal oscillator at 32.768 kHz. All signal processing in active mode is performed at 1 MHz.

To extend device lifetime, we also designed a user-configurable periodic sampling scheme. At the beginning of each recording session, the device can be programmed to continuously record EDA or periodically record 64 s windows of EDA starting at any integer multiple of the on-board real time clock (RTC) minute register. By adjusting the number of minutes between sample windows, users can extend battery life and long-term storage capabilities of the 16-bit microcontroller.

### 2.4. On-Chip Signal Compression

This study implements on-chip signal compression in real time to expand the long-term monitoring capabilities using the available internal memory of a microcontroller. The compression method applied can be subdivided into three stages: (1) computing the wavelet transform of the input EDA signal, (2) sorting the wavelet transform coefficients by magnitude, and (3) encoding the largest coefficients along with their locations. These methods are described below.

#### 2.4.1. Wavelet Transformation of EDA Signal

In our application, Algorithm 1 is used to transform a 1D vector of skin conductance values, x[n], into a 1D vector in the wavelet domain, $W_{4}\left[n\right]$, which is sparse and can be efficiently compressed. The ML-DWT procedure begins by initializing the $A_{0}$ approximation vector (step 4) with 128 samples of skin conductance values, x, representing 64-s of EDA. The Pad() function (step 8) in this procedure improves upon the methods described in Addison [22] to perform the ML-DWT by symmetrically padding the input vector before performing the DWT. Given an input vector A of length *N*, the Pad() function will return a new vector, $A_{\mathrm{pad}}$, which is a copy of A with four additional elements on the left and right sides that mirror the perimeter elements of A, such that:

$$\begin{array}{cc} A & =[x_{0},x_{1},...,x_{n-1}],\\ A_{\mathrm{pad}} & =[...,x_{1},x_{0},|x_{0},x_{1},...,x_{n-1},|x_{n-1},x_{n-2},...]. \end{array}$$

The length of $A_{\mathrm{pad}}$ is N+8, which results in two additional coefficients being generated (one coefficient on each end) during the DWT. This step prevents the generation of large coefficients at the signal borders that can lead to reconstruction errors if omitted in the compression process [41]. Other signal extension modes were considered (no-padding, zero-padding, and periodic-padding), but none led to lower reconstruction errors of the compressed EDA signal than symmetric extension. The symmetrically padded vector, $A_{\mathrm{pad}}$, is then input into the WT() function (step 10) to produce the approximation coefficients (step 33) and the detail coefficients (step 34) at the next transformation level using the ML-DWT algorithm described in Equations (3) and (4). Each $A_{L}$ and $D_{L}$ are half of the length of $A_{\mathrm{pad}}$ due to dyadic scaling factor of 2i. This process is recursively applied four times to generate the level-4 approximation and detail coefficients, $A_{4}$ and $D_{4}$. The band passing nature for each transform level is shown in Figure 2 where short-term (’high-frequency’) variations are captured in the lower level transformations and long-term (’low-frequency’) variations are retained in the higher transform levels. The output of the ML-DWT algorithm is a 1D vector of wavelet coefficients, $W_{4}$, containing the highest approximation coefficients, $A_{4}$, and all detail coefficients, $D_{4}-D_{1}$. The coefficients in $W_{4}$ represent a multi-scale wavelet transformation of the original EDA signal and contain all the information needed to completely reconstruct the original signal using the inverse wavelet transformation.

**Algorithm 1** ML-DWT Algorithm

**Input: x             ▹ len(x)=128**

**Output: $W_{4}$          ▹ len($W_{4}$)=145**

**1:** 
**procedure** **ML-DWT**
**2:** 
**        $A_{L}=\left[x_{0},x_{1},...,x_{127}\right]$**
**3:** 
**        $h_{0}=\left[h_{0,0},h_{0,1},...,h_{0,5}\right]$**
**4:** 
**        $h_{1}=\left[h_{1,0},h_{1,1},...,h_{1,5}\right]$**
**5:** 
**    repeat**
**6:** 
**        $A_{\mathrm{pad}}$ =** **Pad** **($A_{L}$)**
**7:** 
**        L=L+1**
**8:** 
**        $A_{L},D_{L}$ =** **WT** **($A_{\mathrm{pad}}$, $h_{0}$, $h_{1}$)**
**9:** 
**    until L=4**
**10:** 
**    $W_{4}\left[0:11\right]=A_{4}$**
**11:** 
**    $W_{4}\left[12:23\right]=D_{4}$**
**12:** 
**    $W_{4}\left[24:43\right]=D_{3}$**
**13:** 
**    $W_{4}\left[44:78\right]=D_{2}$**
**14:** 
**    $W_{4}\left[79:144\right]=D_{1}$**
**15:** 
**    return $W_{4}$**
**16:** 
**end procedure**
 **17:** 
**function** **Pad** **(A)**
**18:** 
**    N=len(A)**
**19:** 
**    M=N+8**
**20:** 
**    $A_{\mathrm{pad}}\left[0:M-1\right]=\left\{0,0,...,0\right\}$**
**21:** 
**    $A_{\mathrm{pad}}=[x_{3},x_{2},x_{1},x_{0},x_{0},x_{1},...$**

**$...,x_{N-2},x_{N-1},x_{N-1},x_{N-2},x_{N-3},x_{N-4}]$**
**22:** 
**    return $A_{\mathrm{pad}}$**
**23:** 
**end function**
 **24:** 
**function** **WT** **($A_{L}$, $h_{0}$, $h_{1}$)**
**25:** 
**    $N=len(A_{L})$**
**26:** 
**    M=N/2**
**27:** 
**    $A_{L+1}\left[0:M-1\right]=\left\{0,0,...,0\right\}$**
**28:** 
**    $D_{L+1}\left[0:M-1\right]=\left\{0,0,...,0\right\}$**
**29:** 
**    for i=0 to M−1 do**
**30:** 
**        for j=0 to 5 do**
**31:** 
**           $A_{L+1}\left[i\right]=A_{L+1}\left[i\right]+h_{0}\left[j\right]A_{L}\left[2i+j\right]$**
**32:** 
**           $D_{L+1}\left[i\right]=A_{L+1}\left[i\right]+h_{1}\left[j\right]A_{L}\left[2i+j\right]$**
**33:** 
**        end for**
**34:** 
**    end for**
**35:** 
**    return $A_{L+1},D_{L+1}$**
**36:** 
**end function**



#### 2.4.2. Sorting Wavelet Coefficients

The $W_{4}$ vector has many values near zero and is considered sparse. This implies that only a few wavelet coefficients play a considerable role in reconstructing the original EDA signal during the inverse wavelet transformation. This study compresses the EDA signal by leveraging the sparsity of the ML-DWT to create a subset, $\hat{W_{4}}$, that encodes only the largest *K* coefficients of $W_{4}$, along with their positions, using less memory than required to store the original signal. Essentially, $\hat{W_{4}}$ is an approximation of $W_{4}$ composed only of its largest *K* coefficients where lower values of *K* lead to higher compression ratios.

The Compression procedure described in Algorithm 2 begins by sorting the largest *K* coefficients of $W_{4}$ where *K* is defined as the desired number of $W_{4}$ coefficients to retain. The ARGSORT() function returns a list of sorted indices (LSI) that represents the top *K* coefficients of the $W_{4}$ vector. The LSI and $W_{4}$ vectors are then input into the ENCODE() function (step 3) to encode the value and position of the top K coefficients of $W_{4}$ into a block of memory, as shown in Table 1.
**Algorithm 2** ML-DWT Compression**Input: $W_{4}$, *K*****Output: $\hat{W_{4}}$****1:** **procedure****Compression****($W_{4}$, *K*)****2:** **    LSI =****argsort****($W_{4}$, *K*)****3:** **    $\hat{W_{4}}$ =****encode****($W_{4}$, LSI)****4:** **    return $\hat{W_{4}}$****5:** **end procedure** **6:** **function****argsort****($W_{4}$, *K*)****7:** **    LSI={}****8:** **    max=0****9:** **    for j=0 to K−1 do****10:** **        for i=0 to 144 do****11:** **           if *i* exists in LSI then****12:** **               continue****13:** **           end if****14:** **           if abs(W[i])>abs(max) then****15:** **               max=W[i]****16:** **               index=i****17:** **           end if****18:** **        end for****19:** **        LSI[j]=index****20:** **        index,max=0****21:** **    end for****22:** **    return LSI****23:** **end function** **24:** **function****encode****($W_{4}$, LSI)****25:** **    for i=0 to 11 do            ▹$A_{4}=W_{4}\left[0:11\right]$****26:** **        $A\left[i\right]=W_{4}\left[i\right]<<4$           ▹ left bit-shift****27:** **    end for****28:** **    for j=12 to K−1 do          ▹D$\in W_{4}\left[12:144\right]$****29:** **        $D\left[j-12\right]=W_{4}\left[LSI\left[j\right]\right]$****30:** **    end for****31:** **    for k∈{0,2,4,6} do****32:** **        A[j] = A[j]|( LSI[j] & 0x000F)****33:** **        A[j+1] = A[j+1]|((LSI[j]>>4) & 0x000F)****34:** **    end for****35:** **    return $\hat{W_{4}}$****36:** **end function**

#### 2.4.3. Encoding Wavelet Coefficients

We create a custom encoding of the compressed wavelet coefficients to optimize memory usage for the application. The $A_{4}$ coefficients represent the low-pass filter of the skin conductance signal and are represented using unsigned, 12-bit values since all skin conductance values are positive. The MSP430 is based on a 16-bit architecture so the $A_{4}$ coefficients can be encoded in the 12 most significant bits within a 16-bit memory register, shown in the ENCODE() function of Algorithm 2 (step 25). The detail coefficients can be negative and are represented using a signed, 8-bit integer (step 28). The addresses of each *D* coefficient, D.addr, range from 12–144 and are encoded with 8-bits. The D.addr values are split into 2, 4-bit segments where they can be stored alongside the $A_{4}$ coefficients (steps 31 and 32), as shown in Table 1. Since the $A_{4}$ coefficients are always in the top 12 largest coefficients, there is no need to store their location if they are encoded in their original order. The compressed EDA of $\hat{W_{4}}$ in Table 1 is an optimized data structure for a 16-bit architecture and helps extend long-term monitoring capabilities on this low-resource device.

**Figure 5:**
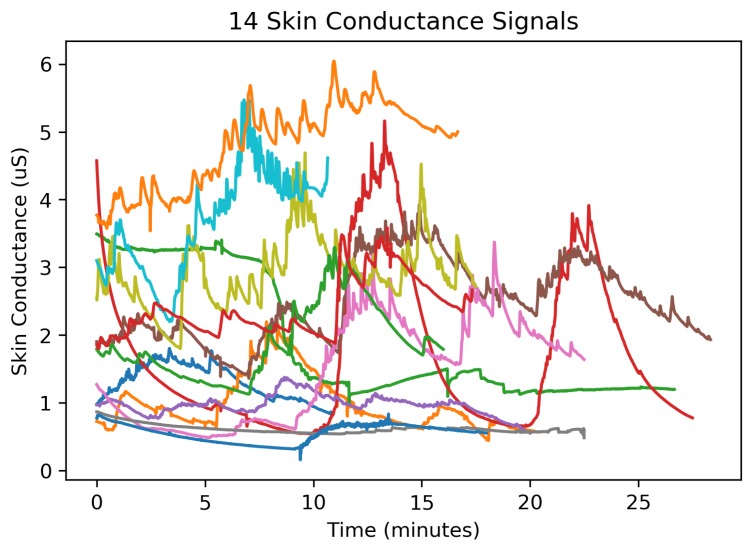
The collection of electrodermal activity signals used to evaluate the compression distortion. These 14 EDA signals were subdivided into 253 segments each representing 64 s of EDA.

Encoding the $W_{4}$ coefficients was optimized by determining the expected range and sign of wavelet coefficients produced from applying the ML-DWT procedure on a collection of EDA signals shown in Figure 5. These EDA signals were recorded during a stress induction protocol conducted across 14 participants (7 male and 7 female) with ages ranging from 24–36 years of age (average age: 27.6 year; median age: 26.5 year; std: 3.57 year). These participants were recruited by email and fliers for an Institutional Review Board (IRB) approved protocol to evaluate the performance an early prototype of the developed system.

Each participant wore the EDA sensor on their right wrist and remained in a seated position while being subjected to a series of tests known to simulate every day stressors [42,43,44,45]. Each stress induction test began with 10 min of relaxation to establish a baseline EDA measurement without stress. When the initial rest period was over, the participant was exposed to a stress induction period lasting 4 min. There were three different stress induction methods: an auditory startle (periodically dropping a textbook on the floor while the participant sat quietly with their eyes closed), a mental arithmetic test (counting backwards from 500 in steps of 7), and public speaking (reciting William Faulkner’s acceptance speech for the Nobel Prize in front of laboratory staff). Each stress induction period was followed by a 5 min period of rest. The order of stress induction tests were randomized and participants conducted anywhere from one to two stress induction tests (with 5 min of rests in between), depending on their willingness to participate in the full experiment. EDA data recordings were visually inspected and data segments having ’flat-line’ skin conductance values below 0.01μS, assumed as having poor or no electrode contact with the skin, were removed from the dataset (ex. data collected before the electrodes are attached to the body). In this way, the compression performance and signal distortion of the new ML-DWT compression algorithm is analyzed only from quality EDA signals recorded in a controlled environment during a stress-induction protocol. These signals were filtered using a Chebyshev type-II filter (0.6 Hz passband at 3 dB; 0.9 Hz stopband at 74 dB) before being compressed. All EDA signals were subdivided into 64 s windows for the evaluation, resulting in 253 independent EDA signals. These recordings are mainly used to characterize the expected performance of an EDA compression algorithm when applied across a population experiencing relaxation and induced stress.

Before performing the ML-DWT, the skin conductance values in x are converted from units of 1μS to units of 0.01μS so that the $W_{4}$ coefficients can be cast from Q15.16 format (1 signed bit; 15 integer bits; 16 fractional bits) into signed, 16-bit integers (Q15.0) and maintain a resolution of ±0.01μS. Table 2 summarizes the minimum memory requirements to encode the $W_{4}$ coefficients by computing the maximum coefficient value in binary notation (bitwidth = $log_{2}\left(max(|X|)\right)$ for $X\in \{A_{4},D_{4},D_{3},D_{2},D_{1},\mathrm{LSI}\}$).

**Figure 6:**
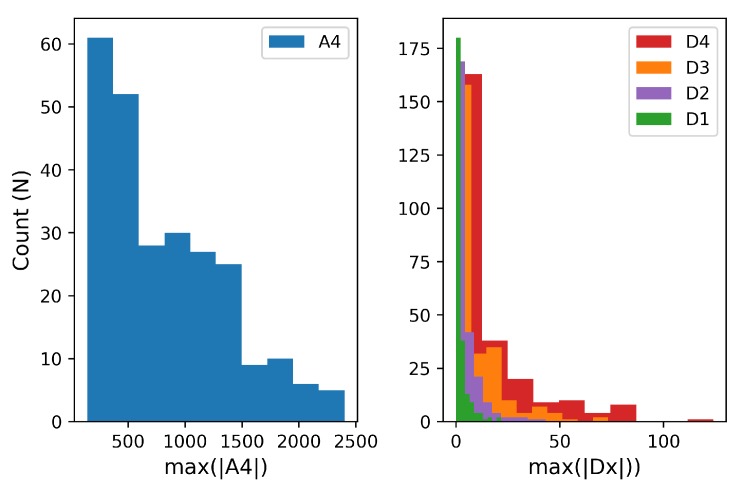
The distribution of wavelet coefficient values for the approximation vector, $A_{4}$, and detail coefficients, $D_{4}-D_{1}$, that compose the 1D wavelet transformation, $W_{4}$. A total of 253 EDA signals were transformed using the ML-DWT algorithm and, for each signal, the maximum magnitude of the wavelet coefficient was recorded. This distribution is used to define the maximum bits required to store the wavelet coefficients.

The magnitudes for each ML-DWT coefficient computed from the EDA signals of Figure 5 are shown in Figure 6. This histogram was used to determine the maximum bits needed to represent the $W_{4}$ coefficients in binary notation. The final required bitwidths to store the $W_{4}$ coefficients and the LSI are summarized in the last column of Table 2 which defines the encoded data structure of Table 1.

### 2.5. Reconstruction

The compressed EDA signal is downloaded to an external device via UART communication for reconstruction. The original EDA signal, x, is reconstructed from $W_{4}$ by populating an empty $W_{4}$ vector with the coefficients retained in $\hat{W_{4}}$ and filling the remaining values with zeros. The symmetric signal extension described earlier was designed to use Python’s PyWavelet library (https://github.com/PyWavelets) for reconstructing the EDA signal from $W_{4}$ using the inverse DWT function, pywt.waverec().

### 2.6. Evaluation and Performance Metrics

#### 2.6.1. Compression Ratio

The compression ratio defines the memory savings achievable with our ML-DWT implementation and is expressed as:

(5) $$CR=\frac{N_{x}}{N_{wt}+N_{i}},$$

where $N_{x}$, $N_{wt}$, and $N_{i}$ are the number of bits used to encode the EDA signal, the wavelet transform coefficients, and $W_{4}$ indices, respectively. Each EDA sample is represented using 32-bit fixed point floats (Q15.16) at a sample rate of 2 Hz, leading to an input data rate of 64 bits/s.

#### 2.6.2. Compression Distortion

Lossy compression inevitably distorts the original signal during reconstruction when signal energy of the ML-DWT is omitted. We use the percent root mean square difference (PRD) [26,46,47] to evaluate the distortion of the reconstructed EDA signals, which is defined as:

(6) $$PRD\left(\%\right)=\frac{\sqrt{\sum_{i=0}^{N-1}{\left(x\left(i\right)-\hat{x}\left(i\right)\right)}^{2}}}{\sqrt{\sum_{i=0}^{N-1}{\left(x\left(i\right)\right)}^{2}}}\times 100.$$

The Root Mean Square Error (RMSErr) is also computed to enable a performance comparison between our wavelet-based compression method and a compressive sensing method presented by Chaspari et al. [10]:

(7) $$RMSErr\left(\mu S\right)=\sqrt{\frac{1}{N}\sum_{i=0}^{N-1}{\left(x\left(i\right)-\hat{x}\left(i\right)\right)}^{2}}.$$

In both equations, x and $\hat{x}$ represent the original and reconstructed signals, respectively, and *N* is the length of the uncompressed signal.

#### 2.6.3. Energy Compaction

The energy compaction of the ML-DWT was evaluated by calculating the percentage of total signal energy (%Energy) contained within each approximation and detail coefficient vector of $W_{4}$ by:

(8) $$\%Energy=\frac{\sum_{i=0}^{n-1}c{\left[i\right]}^{2}}{\sum_{i=0}^{N-1}W_{4}{\left[i\right]}^{2}}\times 100,$$

$$c\in \left\{A_{4},D_{4},D_{3},D_{2},D_{1}\right\};n=length\left(c\right);N=145,$$

where c is an approximation or detail coefficient vector of length *n* and $W_{4}$ is the entire multilevel wavelet transformation vector of length *N*.

#### 2.6.4. EDA Feature Reconstruction Errors

We hypothesize that, since a majority of signal energy is contained in a small number of coefficients, the EDA signal is well-suited for being compressed without significant loss of features associated with the signal. We evaluated this by extracting common EDA features shown in Figure 1 from the original and reconstructed signals and comparing the extracted features as a function of the CR.

For the phasic EDA features, the sum of the skin conductance response (SCR) amplitudes (Sum_Amp) and sum of the SCR durations (Sum_Dur) and their product, the Sum of the Area (Sum_AUC), were computed for each 64 s EDA signal using the third party algorithms provided by Taylor et al. [48].

For each of the 253 EDA signals, the relative error (RE) for a given feature, *f* = (Sum_Amp, Sum_Dur, or Sum_AUC), was computed as follows:

(9) $$RE\left(f\right)=\frac{\sum_{i=1}^{253}f_{recon,i}-\sum_{i=1}^{253}f_{orig,i}}{\sum_{i=1}^{253}f_{orig,i}},$$

where $f_{orig}$ and $f_{recon}$ are features extracted from the original and reconstructed EDA signals, respectively. Additionally, the tonic features extracted from the EDA signal were the Skin Conductance Level (SCL or mean), minimum, maximum, and standard deviation for each EDA signal. The absolute error in (μS) between the original and reconstructed signal was used to evaluate the distortion.

## 3. Results

**Figure 7:**
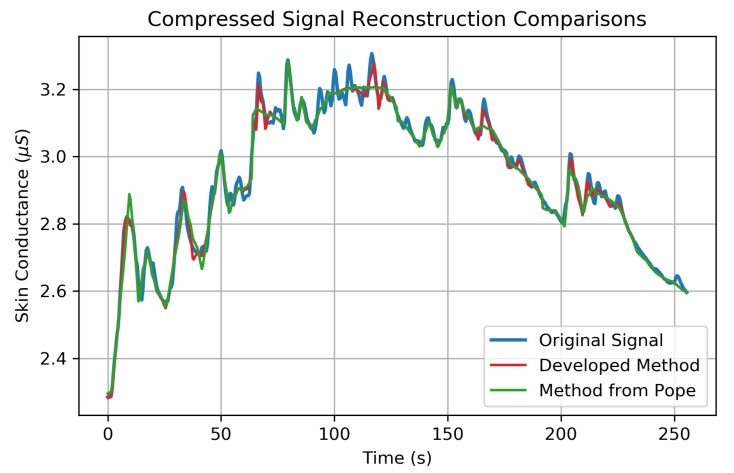
An EDA signal is shown in blue along with a reconstructed EDA signal using the newly developed compression method in red. The previous compression method from Pope et al. 2018 [9] is shown in green for comparison. The original signal is compressed from a data rate of 64 bits/s to 3.75 bits/s in both methods (CR = 17.1×) (The input data rate in [9] used a signed 16-bit representation of skin conductance values instead of the more appropriate 32-bit float representation used here and in [10]. Therefore, the CRs reported in [9] should be doubled for comparison.) The developed method in red is able to encode 18 total $W_{4}$ coefficients, whereas the previous method in green is only capable of encoding the top 14 coefficients leading to a 31.8% improvement in root mean square error (RMSErr). Both reconstructions are composed of four, 64-s compression/decompression cycles spliced together.

A reconstructed signal from the developed compression method is shown in red in Figure 7, along with the original EDA signal in blue. The compression method used in Pope et al. [9] in green is shown to visually compare the reconstruction quality between the two methods. In this figure, both methods compress 64-s windows of EDA signals, consisting of 512 bytes each, into 30-byte encodings of $\hat{W_{4}}$ (CR = 17.1×). In this way, any observable improvement in signal reconstruction quality is related to improvements of the algorithm’s compression efficiency at encoding $W_{4}$ information into $\hat{W_{4}}$. The newly developed method shown in Figure 7 is capable of encoding the top 18 coefficients of $W_{4}$—as opposed to only the top 14 coefficients for the method shown in green from [9]. This improved encoding scheme in Algorithm 2 improves the RMSErr by 31.8% (from 0.0274μS to 0.0208μS) at the same compression ratio.

### 3.1. Compression Performance

**Figure 8:**
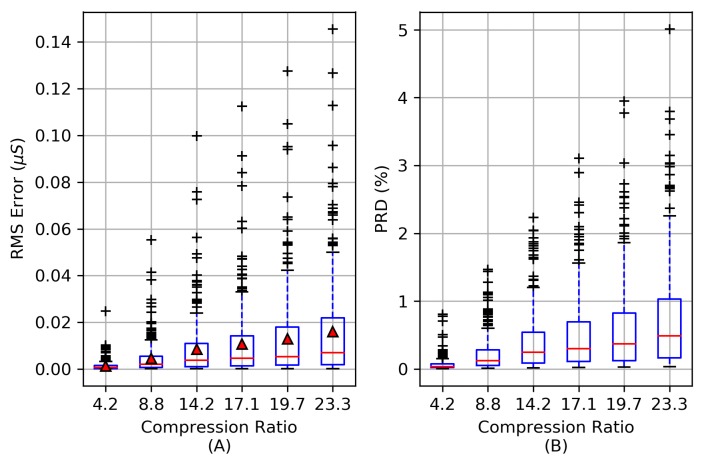
The root mean square error (RMSErr) is shown in (**A**) of 253 EDA signals at a range of compression ratios. The mean RMSErr at each compression ratio is indicated by red triangles. The percent root mean square difference (PRD) distortion in (**B**) is minimal for CRs below 14.2× while the upper quartile range remains below 1% for CRs up to 19.7×.

The distortion of the reconstructed signal is evaluated using the RMSErr and PRD distortion metrics from Equations (6) and (7) to evaluate the quality of the compression process. Figure 8A shows the RMSErr for each reconstruction over a range of compression ratios based on the 253 EDA signals from Figure 5. For CRs up to 23.3×, the RMSErr is below 0.023μS for 75% of all signals evaluated and the average RMSErr is no greater than 0.016μS. For CRs up to 23.3×, the PRD is below 1.1% for 75% of the EDA signals evaluated. As the CR exceeds 23.3×, the PRD rapidly increases as coefficients from the $A_{4}$ vector are omitted.

The ML-DWT transformation tends to compact signal energy into the higher coefficient vectors, leading to a sparse $W_{4}$ vector. This energy compaction leads to >99% of the total signal energy (%Energy) being packed into the $A_{4}$ coefficient vector across a sample of 253 unique EDA signals (Table 3). This allows the original signal to be compressed and reconstructed using very few wavelet coefficients, as shown in Figure 7. The %Energy for each coefficient vector in Table 3 was computed for all 253 EDA signals and shows that a majority of signal energy in the wavelet domain can be retained with very few wavelet coefficients.

### 3.2. EDA Feature Performance

**Figure 9:**
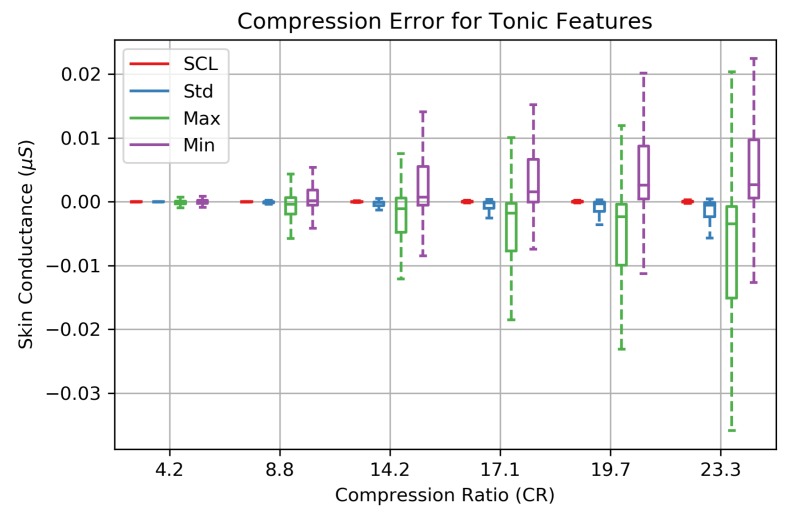
The absolute reconstruction errors of four EDA features computed on 253 EDA signals that were collected during in-laboratory stress tests. The SCL (EDA mean) and standard deviation are hardly effected by compression. While the low-passing filtering effect of compressing the 1D array of wavelet coefficients introduces larger error on the EDA maximum and minimum at higher compression ratios, error within the interquartile remains below 0.015 μS for CRs up to CR = 23.3×.

The features of the EDA signal are not equally impacted at increasing compression ratios. The tonic features are preserved quite well throughout the range of CRs (Figure 9). SCL and standard deviation (Std) feature errors are <0.01 μS up to a CR of 23.3× and are negligible. Errors associated with the EDA maximum (Max) and minimum (Min) are effected more at higher compression ratios. The omission of detail coefficients at higher CRs has a low-pass filtering effect which moves the EDA signal Max and Min towards the mean SCL.

**Figure 10:**
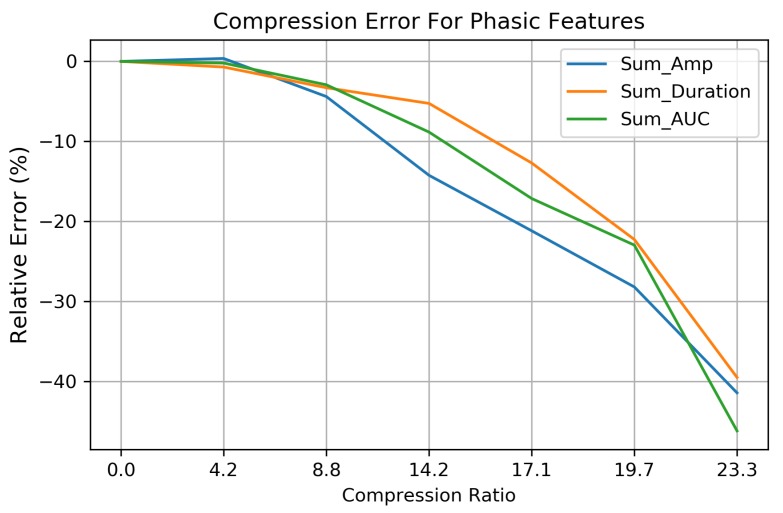
The relative error for the phasic EDA features—Sum of Area Under the Curve, Sum of SCR amplitudes, and the Sum Duration over 253 EDA signals. Reconstruction errors are increased significantly above compression ratios of 8.8×, due to the loss of low-amplitude SCRs not being during the compression process.

Features related to phasic EDA (Sum_AUC, Sum_Amp, and Sum_Dur) are more sensitive to compression and experience greater errors at higher CRs, as shown in Figure 10. The relative errors for these features (based on Equation (9)) show that the phasic features can be preserved quite well up to a CR of 8.8× with a relative error <5.0%. Above this CR, phasic feature errors increase, leading to a 28% relative error when CR = 19.7×. Compression above this point begins to filter out SCRs of increasingly larger amplitudes—leading to relative errors exceeding 75% for the Sum_AUC, Sum_Amp, and Sum_Duration features as less information encoded within the detail coefficients is included in the compressed signal.

### 3.3. Sensor Performance

**Figure 11:**
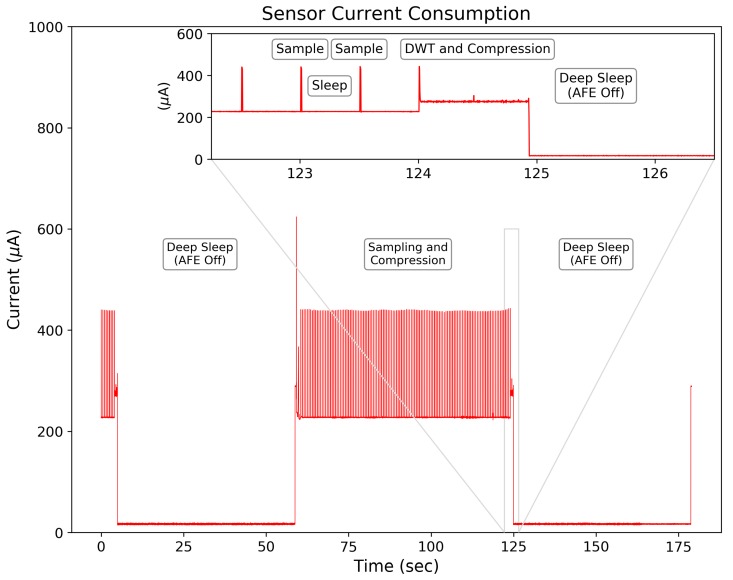
Measured current consumption for the entire system across operational modes with a supply voltage of 2.8 V. The average current draw is 16.6 μA during deep sleep mode, 232 μA for sampling the EDA signal, and 280 μA while processing the ML-DWT.

The designed system has high energy efficiency, consuming 655μW for continuous EDA signal sampling, processing, and recording. Figure 11 shows the current consumption between a 64 s sampling window (232 μA) and deep sleep modes (16.6μA). These low power modes provide large energy savings when used in combination with periodic sampling method discussed previously.

The processing of the ML-DWT, compression encoding, and data storage of the 128 sample EDA signal occurs within 0.92 s and requires an average current of 280μA. This provides efficient end-to-end processing speed at low current consumption, making it competitive with traditional peripheral storage devices and wireless transmission methods used for data storage—especially given the relatively low clock rate of the MSP430 at 1 MHz.

**Figure 12:**
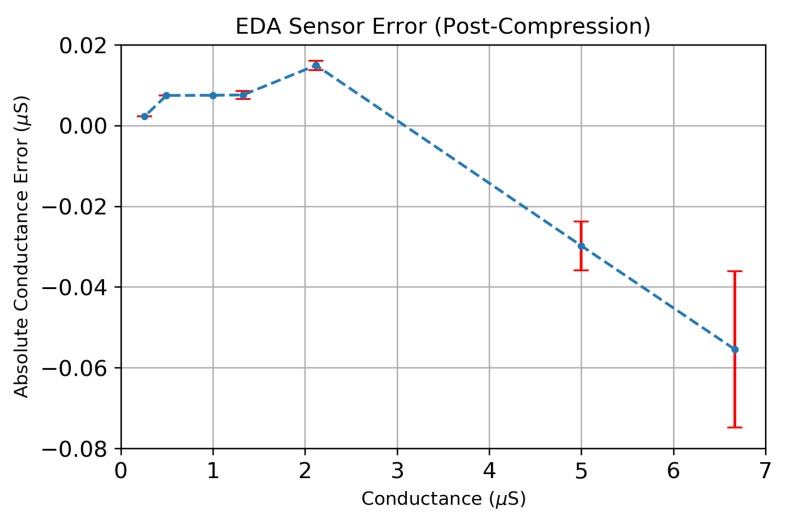
The absolute error of the EDA sensor across a range of conductance values using the compression ratio (CR = 17.1×). Each error measurement consists of 100 measurements of a known, fixed resistor having a conductance equal to G=1/R.

We evaluated the accuracy of the EDA sensor’s analog front end on a range of fixed resistance values, as shown in Figure 12. The series of fixed resistors ranging 150 kΩ–3.88 MΩ were measured to determine the EDA sensor’s conductance measurement error, using the formula G=1/R. Figure 12 shows that the maximum conductance error is <0.075 μS for a range of conductance values between 0.25μS–6.67 μS with a minimum sensitivity of 0.02μS. Each error bar shown consists of 100 individual conductance measurements that were compressed on-board the MSP430 using a CR = 17.1×.

### 3.4. EDA Recording Experience

This ultra-low resource sensor operates as a ‘plug-and-play‘ recorder of electrodermal activity. Each sensor is designed to download data from the device to file on a laptop when the sensor is plugged in for charging. The charging cable also serves as a serial connection to the EDA sensor and a set of Python scripts are used to automatically download data from the device and configure it for the next recording. Each sensor can be programmed to record in ‘Lab’ mode which continuously records EDA without compression at a sample rate of 2 Hz or in ’Field’ mode, in which EDA data is compressed before storing it into internal memory. Higher levels of compression lead to longer recording times at the expense of distorting EDA feature according to the results presented in Figure 9 and Figure 10. Table 4 summarizes the maximum recording duration at a given compression ratio (CR) if saving data into the lower 48 kB of the MSP430FR5969.

## 4. Discussion

In this study, we present an ultra-low resource system for recording the EDA signal at high fidelity entirely within the memory of a 16-bit microcontroller. A multilevel wavelet transformation is implemented on an embedded MCU in real time to create a sparse representation of the EDA signal so that it can be compressed and stored within the internal memory of the MSP430. The system developed here was designed to be a fully autonomous EDA recording device optimized for size and power using minimal resources. The low-resource compression techniques described here could be extended to wireless EDA sensors where size, weight, and power come at a premium.

Many wireless MCUs today, such as Texas Instrument’s CC2650 and Nordic Semiconductor’s nRF51822 (Oslo, Norway), have on-board storage capabilities of 2–32 kB of RAM and 64–256 kB of non-volatile flash memory. At a sample rate of 2 Hz, EDA data could be recorded in raw format for 2.2 h in RAM and 17.8 h in flash memory. The developed method would extend this recording range to 19 h in RAM and 6.3 days using flash memory with very little impact on signal quality, as shown in Figure 7 and Figure 8, to enable long-term monitoring capabilities without the need for continuous wireless connectivity. For example, the ring-based EDA sensor from Moodmetric (Tampere, Finland) requires a very small footprint and currently relies upon an external wireless device to actively record raw EDA data since, “The Moodmetric ring does not store raw data due to limited memory size” [49]. In this use case, our developed methods could (1) enable on-chip storage capabilities to extend physiological recording in moments without wireless connectivity, and (2) reduce the power required to transmit data wirelessly by compressing the EDA signal information.

Implementing the wavelet-compression on-board the MCU is competitive to CS-based methods. Our results in Figure 8 show the mean RMSErr distortion errors of 0.0046 μS at CR = 14.2× and 0.016 μS at CR = 23.3× when compressing 64 s windows of EDA. In comparison, the compressive sampling method from [10], which uses knowledge-based dictionaries as atoms (basis functions) to reconstruct the EDA signal, achieves average RMS errors (approximately) below 0.02 μS with CRs below 17.7×, using 10 s windows for each reconstruction and >12 orthogonal matching pursuit (OMP) iterations per reconstruction. This method would require roughly 72 OMP optimization iterations during the reconstruction of a 64 s EDA signal as opposed to the single inverse DWT required for our wavelet-based method. Therefore, our on-board compression approach may be better suited for applications intending to implement EDA signal reconstructions on another mobile device, such as data visualization on a mobile phone, where efficient and quick processing of EDA signal reconstructions is desired. Although direct comparisons are challenging, a compressed sensing approach does not appear to provide large gains in compression performance compared to our wavelet-based method. The results from Lou et al. [50], who used CS methods based on multi-level wavelet transforms, states that their CS method, “...possesses enough advantage [over on-board compression] in some circumstances e.g., there is a rigid demand on compression time and a loose limit on decompression, or it is not easy to get complete original data.” The work from this study provides support that (1) the compression time of the EDA signal is minimal (0.9 s, shown in Figure 11) compared to the signal length period of 64 s and (2) given the low data rate of the EDA signal, the original data is easy to acquire in real time and could be used for further signal processing. Furthermore, the cost of compressing the original signal on-board the sensor may provide additional signal processing benefits that are not available using CS methods, such as removing ambulatory noise from the EDA signal before compression, to improve the reconstruction performance (although this was not implemented here and would be a topic of future studies).

Using a microcontroller’s internal memory to store physiological data can have useful benefits to power efficiency. A recent review [4] shows that power typically used to transmit (TX) and receive (RX) data in wireless sensor nodes is ∼60 mW (using the TI CC2420) and ∼75 mW when saving data to EEPROM (using the Amtel AT45DB321B flash memory, (Santa Clara, CA, United States)) and suggests that using on-board signal processing and on-board feature extraction could improve energy savings by >20×. Our technique supports this argument by combining on-board signal processing and data storage within a single MCU to achieve storage with an average power consumption of 655μW for long-term monitoring (Figure 11). Compared to saving data to EEPROM, our techniques requires 114× less power during continuous operation (75 mW vs. 655 μW).

Building wearable EDA sensors on ultra-low resource systems can improve power and size for wearable applications, but this approach also comes with limitations. The developed system uses only <64 kB for all program memory, RAM, and data storage which requires meticulous attention to detail when allocating memory for the program (e.g., volatile vs. non-volatile memory, stateful vs. stateless variables, etc.) across various modes of low-power operation on the MSP430. Even though we demonstrate that using a microcontroller’s internal memory to save raw data is clearly more power efficient than transmitting it wirelessly to an external device, there are benefits to wireless systems capable of streaming data in real time for remote processing that should be considered. Another possible limitation of the developed system is that the AFE for the EDA sensor was designed using minimal resources to improve sensor size and power, which also has limitations in the range and linearity of conductivity measurement common to DC-based sensor topologies. This point is discussed in more detail in Appendix A.

We demonstrate the impact that compression has on specific EDA features and recognize that not all features are affected equally at increasing compression ratios. The tonic characteristics of the EDA signal (SCL, SC Min, SC Max, SC Std) tend to be preserved with minimal distortion at higher CRs (Figure 9), while the short term, phasic fluctuations of the EDA signal (SCR features: Sum_Amp, Sum_Duration, Sum_AUC) are lost at higher compression ratios (Figure 10). This suggests that acceptable levels of EDA signal compression are dependent on the features of interest for a given application. This observation can be attributed to the low-pass and band-passing nature of the multi-resolution decomposition where the large-scale (low frequency) tonic signal information is compacted into the higher wavelet transformation levels ($A_{4},D_{4}$, etc.) while small-scale (higher frequency) phasic EDA information is represented in the lower levels of the detail coefficients. Since >99.9% of signal energy is retained within the $A_{4}$ vector (Table 3), our compression technique favors retention of tonic EDA activity, which is evident in the low reconstruction errors in Figure 9.

Removing the external storage requirements of an EDA sensors has many system-level implications when designing wearable biosensors. Sensor size and costs are reduced by eliminating external memory chips, wireless communication ICs, and radio antennas. Reduced power requirements lead to smaller battery sizes. Smaller batteries improve wearability and comfort of a wearable biosensor. Therefore, clear improvements in size, comfort, and cost can be achieved with a well-matched system design for a given application. These low-resource design strategies could be useful in remote monitoring applications (in-home care, primary care, workplace mHealth, etc.) where long-term monitoring is desirable, but wireless connectivity is limited and/or unavailable. The improvements in sensor size presented could be useful in extreme mobile environments, such as military applications or competitive athletics, where sensor weight and form factor have high premiums. This sensor could additionally be integrated with a wireless transmitter to allow for low-power data storage within the MSP430 during periods without wireless connectivity and permit wireless data transmission only at the most opportune times.

## 5. Conclusions

We designed a system to record EDA signals for extended periods of time entirely within a single, low-resource microcontroller having 64 kB of available memory. A multi-resolution wavelet transformation was used to compress the electrodermal activity biosignal in real time to allow for multi-day storage within the microcontroller. We evaluate the effects that compression has on common EDA signal features and show improvements in power and size using these signal processing techniques. Our on-board implementation of data compression is efficient and competitive when compared to other compressive sensing methods for monitoring EDA. Applications of this technology could improve long-term monitoring capabilities of in-home care, primary care, or military applications in environments with infrequent wireless connectivity or sensing modalities where sensor size and weight have a high premium.

## 6. Patents

Halter, R.J., Pope, G.C., “A micro-recording device for physiological signals”, Application Number: 62722520, August 2018. 

## Figures and Tables

**Table 1:** The compressed EDA data structure—$\hat{W_{4}}$.

Bit															
**15**	**14**	**13**	**12**	**11**	**10**	**9**	**8**	**7**	**6**	**5**	**4**	**3**	**2**	**1**	**0**
$A_{4}\left[0\right]$												D[0].addr[0]			
$A_{4}\left[1\right]$												D[0].addr[1]			
$A_{4}\left[2\right]$												D[1].addr[0]			
$A_{4}\left[3\right]$												D[1].addr[1]			
...												...			
$A_{4}\left[10\right]$												D[5].addr[0]			
$A_{4}\left[11\right]$												D[5].addr[1]			
D[1]								D[0]							
D[3]								D[2]							
D[5]								D[4]							
D[x]								D[x].addr[1]				D[x].addr[0]			
:								:				:			

**Table 2:** Wavelet coefficient memory requirements.

WT Level	Max Value	Max Base 2	Bits Required	Sign Bit?	Bitwidth Selected
$A_{4}$	2399.53	11.2285	12	No	12
$D_{4}$	123.881	6.95281	7	Yes	8
$D_{3}$	73.2285	6.1943	7	Yes	8
$D_{2}$	43.2475	5.43454	6	Yes	8
$D_{1}$	21.7329	4.44181	5	Yes	8
LSI	145	7.17991	8	No	8

**Table 3:** Average percent energy of wavelet coefficient vectors.

WT Vector	Mean %Energy	Std
$A_{4}$	99.98%	0.04453%
$D_{4}$	0.01125%	0.02632%
$D_{3}$	0.006232%	0.01582%
$D_{2}$	0.002031%	0.006512%
$D_{1}$	0.0008842%	0.004310%

**Table 4:** EDA recording duration for a given compression ratio (CR) using 48 kB of memory storage.

Compression Ratio (CR)	Recording Duration (hours)
0	0.60
4.20	2.52
8.80	5.28
14.20	8.52
17.10	10.26
19.70	11.82
23.30	13.98

**Figure A1:**
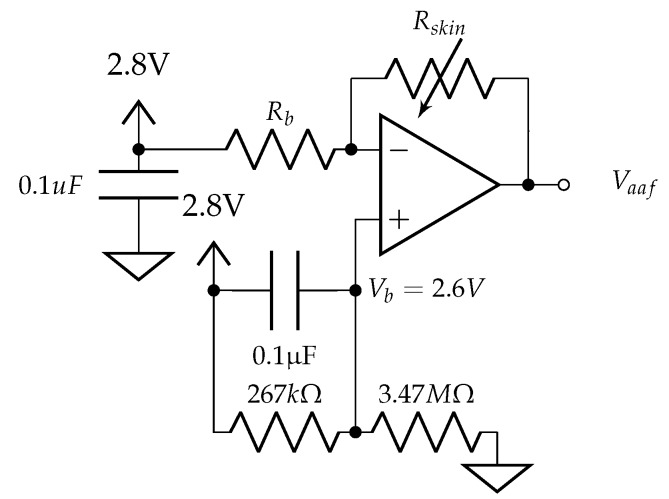
EDA sensor amplifier.

**Figure A2:**
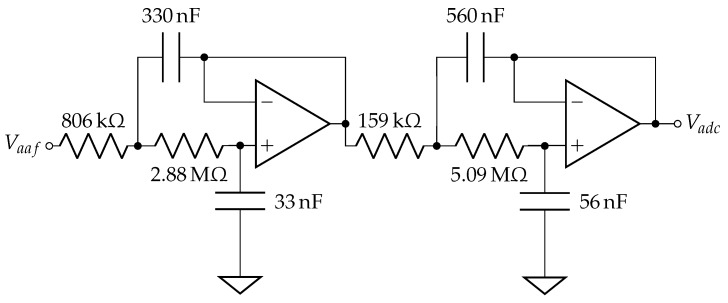
Anti-aliasing filter design.

## Appendix A. EDA Sensor Circuitry

In this topology (Figure A1), the fixed voltage $V_{b}$ creates a 0.2 V voltage drop across resistor $R_{b}$ = 330 kΩ and establishes a quasi-constant current of 0.6 μA through resistor $R_{skin}$, the lumped resistance of the skin and contact electrodes. This topology was specifically designed to measure a fixed range of skin conductivities from 0.25 μS–6.67 μS (although a wider range from 0.01 μS–25 μS may be more appropriate for large populations [51]).

A recent publication from Pabst et al. [52] examines the nonlinear behavior of human skin when measuring skin impedance with low-frequency excitation voltages (0.2 V–1.2 V) and suggests that the EDA measurement itself may be affecting the underlying electrical properties of the skin during measurement when DC excitation voltages are >0.5 V (the standard method). Low-current measurements of EDA have been shown by Yamamoto and Yamamoto [53] to reduce nonlinear affects and, more recently, Pabst et al. [54] mention that further research into the nonlinear behavior of the skin at low current densities is still required. With an electrode area of 0.785 cm${}^{2}$, the developed system has a density of 0.6 μA/0.785 cm${}^{2}$ = 0.764 μA/cm${}^{2}$. The study from [52] observes nonlinear electrical properties of the skin at higher excitation voltages and current levels. While this quasi-constant current circuit topology in Figure A1 may benefit by maintaining low levels of current required for linear EDA measurement, we recognize that the results from [52] suggest AC topologies may provide additional benefits’ linear operation at higher frequencies >0.1 Hz. Alternatively, this circuit topology could be modified to measure EDA using a constant-voltage model by exchanging the positions of $R_{skin}$ and $R_{b}$ and adjusting the reference voltage at $V_{b}$ such that the voltage drop across the skin is within the linear range of operation (0.2 V DC) suggested in [52].

As the skin resistance changes, due to activation of the sympathetic nervous system, the voltage output at node $V_{aaf}$ can be modeled as:

$$V_{aaf}=\frac{R_{skin}}{R_{b}}\left(-0.2V\right)+2.6V.$$

## Appendix B. Analog Low Pass Filter Design

The topology in Figure A2 cascades two Sallen–Key low-pass filters to achieve a 4th order Butterworth filter with a 0 dB passband and a −3 dB cutoff frequency at 1 Hz. The entire AFE was powered by a low-noise voltage regulator at 2.8 V (Texas Instruments, LP5907) that can be powered down when no skin contact is detected.
